# Identification of Candidate Susceptibility and Resistance Genes of Mice Infected with *Streptococcus suis* Type 2

**DOI:** 10.1371/journal.pone.0032150

**Published:** 2012-02-27

**Authors:** Jie Rong, Wei Zhang, Xiaohui Wang, Hongjie Fan, Chengping Lu, Huochun Yao

**Affiliations:** Key Lab of Animal Bacteriology, Ministry of Agriculture, Nanjing Agricultural University, Nanjing, China; Instituto Butantan, Brazil

## Abstract

*Streptococcus suis* type 2 (SS2) is an important swine pathogen and zoonosis agent. A/J mice are significantly more susceptible than C57BL/6 (B6) mice to SS2 infection, but the genetic basis is largely unknown. Here, alterations in gene expression in SS2 (strain HA9801)-infected mice were identified using Illumina mouse BeadChips. Microarray analysis revealed 3,692 genes differentially expressed in peritoneal macrophages between A/J and B6 mice due to SS2 infection. Between SS2-infected A/J and control A/J mice, 2646 genes were differentially expressed (1469 upregulated; 1177 downregulated). Between SS2-infected B6 and control B6 mice, 1449 genes were differentially expressed (778 upregulated; 671 downregulated). These genes were analyzed for significant Gene Ontology (GO) categories and signaling pathways using the Kyoto Encylopedia of Genes and Genomes (KEGG) database to generate a signaling network. Upregulated genes in A/J and B6 mice were related to response to bacteria, immune response, positive regulation of B cell receptor signaling pathway, type I interferon biosynthesis, defense and inflammatory responses. Additionally, upregulated genes in SS2-infected B6 mice were involved in antigen processing and presentation of exogenous peptides, peptide antigen stabilization, lymphocyte differentiation regulation, positive regulation of monocyte differentiation, antigen receptor-mediated signaling pathway and positive regulation of phagocytosis. Downregulated genes in SS2-infected B6 mice played roles in glycolysis, carbohydrate metabolic process, amino acid metabolism, behavior and muscle regulation. Microarray results were verified by quantitative real-time PCR (qRT-PCR) of 14 representative deregulated genes. Four genes differentially expressed between SS2-infected A/J and B6 mice, toll-like receptor 2 (*Tlr2*), tumor necrosis factor (*Tnf*), matrix metalloproteinase 9 (*Mmp9*) and pentraxin 3 (*Ptx3*), were previously implicated in the response to *S. suis* infection. This study identified candidate genes that may influence susceptibility or resistance to SS2 infection in A/J and B6 mice, providing further validation of these models and contributing to understanding of *S. suis* pathogenic mechanisms.

## Introduction

*Streptococcus suis*, a Gram-positive encapsulated coccus, is considered to be an important swine pathogen, which not only causes septicemia but also affects the central nervous system (CNS) and other tissues, leading to meningitis, endocarditis, pneumonia and arthritis [1], [2]. Although 33 serotypes have been recognized on the basis of capsular antigens, serotype 2 is still the most frequently isolated from diseased animals [3]. *S. suis* does not only cause disease in pigs but also affects humans. Human infection with *S. suis* mainly occur in people with occupational exposure to infected pigs or raw pork products and have been reported in different Asian and European countries, as well as in New Zealand, Australia, Argentina and Canada [4], [5], [6], [7].

The pathogenesis of both systemic and CNS infections caused by *S. sius* is poorly understood. To induce clinical disease in swine, it is believed that *S. suis* enter through the respiratory route and remain localized in the tonsils. In humans, however, the route of infection is mainly through skin injuries when bacteria may gain access to the bloodstream, where they disseminate freely or as cell-bound bacteria attached to phagocytes [2] until reaching the CNS. Septicemia and meningitis may be related to an exacerbated or uncontrolled inflammatory response that is also, in the case of meningitis, accompanied by an increase in the permeability or breakdown of the blood-brain barrier [2]. For example, *S. suis* can upregulate expression of adhesion molecules on monocytes, thereby increasing leukocyte recruitment to infection sites and boosting the inflammatory response [8]. It was reported that human and murine monocytes/macrophages recognize the intact *S. suis* or its purified cell wall components through a toll-like receptor 2 (Tlr2)-dependent pathway, with the possible participation of CD14, and release of cytokines and chemokines [9], [10], [11].

Animal models are essential to obtaining a better understanding of pathogenesis of *S. suis*, and mice have been used as an experimental model for evaluation of *S. suis* virulence [12], [13], [14]. Research by Williams *et al.* showed that the behavior of *S. suis* type 2 (SS2) in infected mice resembles that in pigs [12]. Previous research indicated that BALB/c and SS strains of mice are useful as experimental models of SS2 infections in pigs. The type strain and isolates of this *S. suis* type from diseased pigs produce septicemia and meningitis in BALB/c and SS mice inoculated with 10^8^ colony forming units (CFU) of the bacteria and 60 to 100% of these infected mice die. In BALB/c mice that die or develop nervous signs due to SS2 infection, purulent meningoencephalitis, myocarditis, ophthalmitis, labyrinthitis and otitis media were observed [14]. Recently, a hematogenous model of *S. suis* infection in adult CD1 outbred mice was developed by Dominguez-Punaro and colleagues, and this experimental model may be useful for studying the mechanisms underlying sepsis and meningitis during bacterial infection [15]. Their further research demonstrated that A/J mice are significantly more susceptible to *S. suis* infection than C57BL/6 (B6) mice, especially during the acute septic phase of infection [16]. Assessment of susceptibility to *S. suis* using animal models has long been limited to monitoring mortality rates and histopathological studies, but the genetic basis of susceptibility to *S. suis* infection is largely unknown. Therefore, we used Illumina mouse BeadChips in this study to identify alterations in gene expression of mice injected with SS2 strain HA9801. Such whole transcriptome analyses would contribute to future studies of transmission, virulence and pathogenesis of *S. suis*.

## Materials and Methods

### Ethics statement

All animals used in this study, and animal experiments, were approved by Department of Science and Technology of Jiangsu Province. The license number was SYXK(SU) 2010-0005.

### Bacterial strains and culture conditions

SS2 HA9801, originally isolated by our laboratory, is considered a virulent strain [17], [18], [19], [20]. Bacteria were grown overnight on sheep blood agar plates at 37°C, and isolated colonies were inoculated into 5 mL cultures of Todd-Hewitt broth (THB) (Oxoid), which were incubated for 12 h at 37°C with agitation. Working cultures were prepared by transferring 300 µl of the 12 h cultures into 30 mL of THB, which were further incubated for 3–4 h at 37°C with agitation. Late log phase bacteria were washed twice in phosphate-buffered saline (PBS) (pH 7.4). The pelleted bacteria were then resuspended and adjusted to a concentration of 5×10^8^ CFU/mL. The inoculum for experimental infection was diluted in THB to obtain a final concentration of 1×10^8^ CFU/mL. This final suspension was plated onto blood agar to accurately determine the CFU/mL.

### Mice and experimental infection

Specific pathogen-free mice of the B6 and A/J strains were purchased from the Model Animal Research Center of Nanjing University. Female mice of 8–14 weeks of age were acclimated to standard laboratory conditions of a 12-h light/12-h dark cycle with free access to rodent chow and water. A preliminary study was performed to verify the 50% lethal dose (LD50) of the HA9801 strain and to determine the optimal bacterial dose and time points. For the microarray experiment, experimental and mock infections of mice were performed by intraperitoneal inoculations according to the following groups: Five A/J and five B6 mice were each injected with a 200 µL volume of the SS2 HA9801 bacterial suspension (1×10^8^ CFU/mL); Five A/J and five B6 control mice were each injected with a 200 µL volume of the vehicle solution (sterile THB).

### Extraction of peritoneal macrophages

Control and SS2-infected A/J and B6 mice (three in each group) were sacrificed at 9 h post-infection. The peritoneal macrophages were harvested according to a procedure reported elsewhere [21]. Resident peritoneal macrophages were collected from A/J and B6 mice by flushing of the peritoneal cavity with 5 mL ice-cold Hank's balanced salt solution containing 10 U/mL of heparin. Peritoneal cells were plated at a density of 1×10^6^ cells/cm^2^ in RPMI medium supplemented with 10% FBS, and macrophages were left to adhere for 2 h in a humidified atmosphere at 37°C with 5% CO_2_. Non-adherent cells were washed off the plate, and the adherent cells were considered macrophages.

### RNA preparation

The peritoneal macrophages of each mouse were lysed, and total RNA was extracted using Trizol reagent (Invitrogen). The partial RNA from the peritoneal macrophages of each of three mice from each group were pooled to minimize biological variation in gene expression within a group [22]. The left RNA samples were remained for qRT-PCR. One sample of pooled RNA for each group was further purified using an RNeasy Mini kit (Qiagen) according to the manufacturer's instructions and submitted for microarray hybridization. The integrity of the isolated RNA was assessed both before and after pooling by formaldehyde denaturation gel electrophoresis. RNA concentration and purity were determined by spectrophotometry. Only those samples that had an OD260/OD280 ratio of approximately 2.0 and showed no degradation (ratio approaching 2∶1 for the 28S and 18S bands) were used to generate labeled targets.

### Illumina BeadChip gene expression and data analysis

The RNA samples were sent to Biostar Genechip Inc. (Shanghai, China) for microarray hybridization. The pooled RNA sample from each group was hybridized to one Illumina mouse Genome Beadchip Array (catalog number 5022612022, Mouse WG-6_V2, Illumina). Therefore, four BeadChips were used in total, one for each of the A/J and B6 infected and control mice groups. Biotin-labeled cRNA preparation and hybridization were performed as described previously [23]. The arrays were scanned on an Illumina BeadStation 500 System and the hybridization data analyzed using Illumina BeadStudio software. The following filtering criteria were used for selection of differentially expressed genes: positive gene in either test or control, and test DiffScore ≥+20 or ≤−20. The differentially expressed genes were selected by comparing the following groups: SS2-infected A/J vs. SS2-infected B6; control A/J vs. control B6; SS2-infected A/J vs. control A/J; SS2-infected B6 vs. control B6. All data were MIAME compliant, and the raw data has been deposited in ArrayExpress database along with normalized data. The accession assigned is E-MTAB-745.

### Gene ontogeny (GO) category and pathway analysis

**Figure 1:**
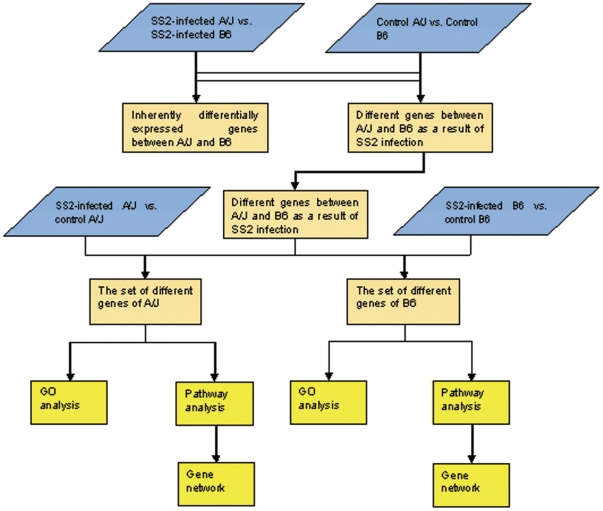
The process of treatment of four groups of data for GO, pathway and gene network analysis. (a) The differentially expressed genes between control A/J and control B6 mice were eliminated from those between SS2-infected A/J and SS2-infected B6 mice. (b) The remain of differential genes between SS2-infected A/J and SS2-infected B6 were intersected with differentially expressed genes between SS2-infected A/J and control A/J mice. (c) The remaining set of differentially expressed genes were analyzed for inclusion in GO categories and pathways. The same process was carried out with the differentially expressed genes between SS2-infected B6 and control B6 mice.

The differentially expressed genes between SS2-infected A/J and control A/J mice were intersected with those between SS2-infected A/J and SS2-infected B6. The same process was carried out with the differentially expressed genes between SS2-infected B6 and control B6. The differentially expressed genes between control A/J and control B6 mice were eliminated, as they were considered the genes that were inherently different between A/J and B6 mice. The remaining set of differentially expressed genes were analyzed for inclusion in GO categories and pathways. The concrete treatment for four groups of data is presented in Figure 1. Categorization in significant biological processes was performed using tools of the Gene Ontology project (http://www.geneontology.org) [24]. The test of statistical significance considers the number of differentially expressed genes found in each category compared with the total number of genes in the category represented on the chip. The pathway analysis was carried out using the Kyoto Encyclopedia of Genes and Genomes (KEGG) database [25]. Two-sided Fisher's exact test and *χ* ^2^ test were used to classify the GO category and pathway, and the false discovery rate (FDR) was calculated to correct the *P* value. *P* value<0.05 and FDR<0.05 were used as a threshold to select significant GO categories and KEGG pathways.

### Gene network analysis

The gene network analysis of the differentially expressed genes involved in significant pathways was carried out using the KEGG database. Interactions of genes in the database were analyzed, and gene networks were established. The degree of connectivity was used to evaluate the role of genes in the network.

### Confirmation of BeadChip results by quantitative real-time PCR (qRT-PCR)

Total RNA from each of three mice of each group was treated as same as the pooled RNA for BeadChips and the integrity was assessed. One microgram of total RNA from each of three mice of each group was used in a reverse transcription reaction of 20 µL total volume to synthesize first strand cDNA using Transcriptor First Strand cDNA Synthesis Kit (Roche) according to the manufacturer's instructions. According to the relative researches and network analysis results, the specific genes were selected for verification. Primers were designed to amplify sequences of 75–250 base pairs (bp) (Table 1). For real-time PCR, the 7300 Real-Time PCR System (ABI) and FastStart Universal SYBR Green Master (Roche) were used. Each reaction contained 1 µL cDNA template and 9 µL SYBR Green Master. Amplification conditions were 95°C for 10 m, followed by 40 cycles of 95°C for 15 s and 60°C for 60 s. Each sample and no template controls were run in duplicate. Glyceraldehyde-3-phosphate dehydrogenase (*GAPDH*) was also amplified under the same conditions as the internal control to normalize reactions. After completion of the PCR amplification, the relative fold change after infection was calculated based on the 2^−ΔΔCT^ method [26].

**Table 1:** Primers for selected genes analyzed using qRT-PCR.

Acronym	Gene name	Primer sequences (5′-3′)	GenBank number	Product size (bp)
*Itgb2*	integrin beta 2	GGCTGGATGCCATAATGCAAG AAGCCATCGTCTGTGGCAAAC	NM_008404.4	94
*Itgal*	integrin alpha L	GGAATGACGCTGGCAACAGA AGGTAGCAGAGGCCACTGAGGTAA	NM_008400.2	107
*Pdpk1*	3-phosphoinositide dependent protein kinase-1	GCAGGACTCCCACCATTCAGA GCCTAAACGCTTTGTGGCATC	NM_001080773.1	150
*Icam2*	intercellular adhesion molecule 2	CATCTCGGAGTACCAGATCCTTGAA GCAGTATTGACACCACCACGATG	NM_010494.1	86
*Irf9*	interferon regulatory factor 9	TGCTGCCCAGCAATAAGTGTG CCAGAAATGTAGGGTTGCTTGGA	NM_008394.3	119
*Stat1*	signal transducer and activator of transcription 1	GGCTGCCGAGAACATACCAGA CCAGTTCGCTTAGGGTCGTCA	NM_009283.2	138
*Stat2*	signal transducer and activator of transcription 2	TGCAGCGAGAGCACTGGAA CATTGGCAGGATGCTCTGTGA	NM_019963.1	137
*Socs2*	suppressor of cytokine signaling 2	CTGCGCGAGCTCAGTCAAAC TAGTCGGTCCAGCTGACGTCTTAAC	NM_007706.3	163
*Tlr2*	toll-like receptor 2	GGAGCATCCGAATTGCATCAC TTATGGCCACCAAGATCCAGAAG	NM_001905.3	116
*Tnf*	tumor necrosis factor	GTTCTATGGCCCAGACCCTCAC GGCACCACTAGTTGGTTGTCTTTG	NM_013693.2	175
*Mmp9*	matrix metallopeptidase 9	GCCCTGGAACTCACACGACA TTGGAAACTCACACGCCAGAAG	NM_013599.2	85
*Ptx3*	pentraxin related gene	ATGACTACGAGCTCATGTATGTGAA TGAACAGCTTGTCCCACTCC	NM_008987.3	120
*Itga5*	integrin alpha 5	GTTTCAGGCTGCGCTGTGAG CTGGTAGGGCATCTTCAGAGCTTC	NM_010577.3	161
*Il10*	interleukin 10	GACCAGCTGGACAACATACTGCTAA GATAAGGCTTGGCAACCCAAGTAA	NM_010548.1	77
*GAPDH*	glyceraldehyde-3-phosphate dehydrogenase	ATGCCTGCTTCACCACCTTCT ATGTGTCCGTCGTGGATCTGA	NM_008084.2	81

## Results

### Determination of LD50 of strain HA9801 and experimental infection for microarray analysis

The LD50 of strain HA9801 was determined by injecting mice with various doses, and mortality was monitored until 7 days post-infection. The mortality for A/J mice injected with a dose of 10^7^ CFU between 12 h and 96 h was 50% (Table 2). The clinical signs of disease of A/J mice were depression-like behavior, rough appearance of hair coat and swollen eyes [15]. Mice exhibiting extreme lethargy were considered moribund and were humanely euthanized. All of B6 mice injected with a dose of 10^8^ CFU survived, although they all died when injected with a high dose of 10^9^ CFU(data not shown). Control mice showed no death or clinical signs of disease during the 7 days of observation. As B6 are known to be more resistant to *S. suis* infection than A/J mice, the results were in complete accordance with previous research [16]. On the basis of these results, experimental mice were injected with 2×10^7^ CFU for the microarray experiment. At 9 h post-infection, six infected mice (three A/J mice and three B6 mice) and six control mice (three A/J mice and three B6 mice) were selected for analysis.

**Table 2:** LD50 of strain HA9801 on A/J mouse.

Strain	Infection dose(CFU)	Amount of mouse (No)	Mortality/total	LD50(CFU)
HA9801	10^8^	6	4/6	1.61×10^7^
	10^7^	6	3/6	
	10^6^	6	1/6	
	10^5^	6	1/6	
Control		3	0/3	

### Microarray analysis

We hypothesized that gene expression would vary in response to SS2 infection in the peritoneal macrophages of B6 and A/J after intraperitoneal inoculation. To identify such genes, studies were performed using Illumina BeadChip microarrays, which revealed 3,692 differentially expressed genes in peritoneal macrophages between A/J and B6 mice as a result of SS2 infection. (The differentially expressed genes between control A/J and control B6 mice were used to exclude those genes which were thought to be inherently different between A/J and B6 mice.) Between the SS2-infected A/J and control A/J mice, 2646 genes were identified to be differentially expressed, of which 1469 genes were upregulated and 1177 genes downregulated. Between the SS2-infected B6 and control B6 mice, 1449 genes were differentially expressed, of which 778 genes were upregulated and 671 genes downregulated. The differentially expressed genes of the four groups and the group of 3,692 differentially expressed genes are summarized in Table S1.

### GO categorization

**Figure 2:**
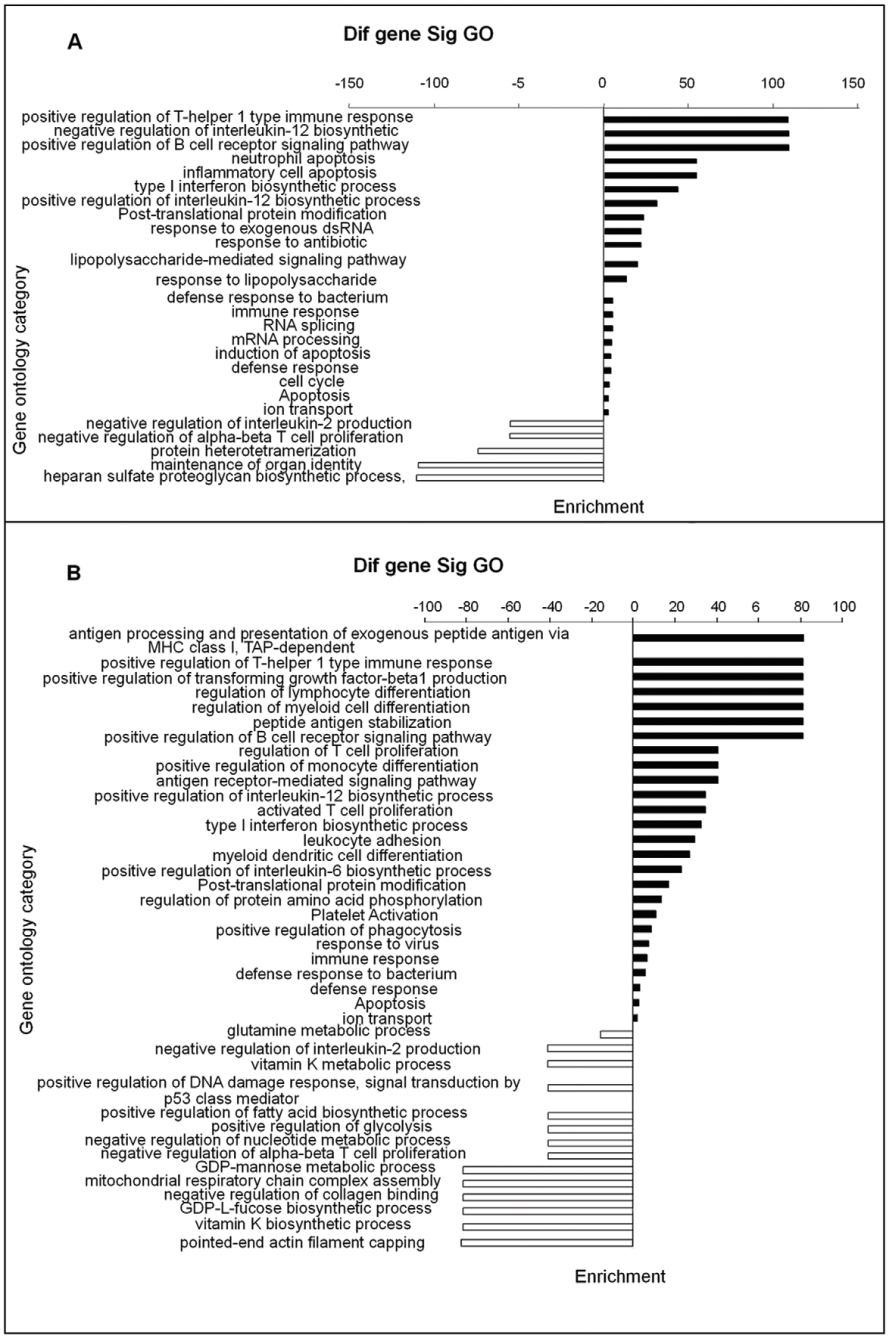
GO categories of biological processes for significantly differentially expressed genes. (A) between SS2-infected A/J and control A/J mice and (B) between SS2-infected B6 and control B6 mice. *P* value<0.05 and FDR<0.05 were used as thresholds to select significant GO categories.

The differentially expressed genes of A/J and B6 mice after infection with strain HA9801 were classified into different functional categories according to the Gene Ontology project for biological processes. The main GO categories for significantly upregulated genes between SS2-infected A/J and control A/J mice were positive regulation of T-helper 1 type immune response, regulation of interleukin-12 biosynthetic process, positive regulation of B cell receptor signaling pathway, type I interferon biosynthetic process, defense response to bacteria, immune response, ion transport and inflammatory cell apoptosis. The main GO categories for significantly downregulated genes between SS2-infected A/J and control A/J mice included negative regulation of interleukin-2 production, negative regulation of αβ-T cell proliferation, protein heterotetramerization and heparan sulfate proteoglycan biosynthetic process (Fig. 2A).

The primary GO categories for significantly upregulated genes between SS2-infected B6 and control B6 mice were antigen processing and presentation of exogenous peptide antigen, positive regulation of T-helper 1 type immune response, peptide antigen stabilization, positive regulation of B cell receptor signaling pathway, regulation of lymphocyte differentiation, positive regulation of monocyte differentiation, antigen receptor-mediated signaling pathway, positive regulation of interleukin-12 biosynthetic process, type I interferon biosynthetic process, platelet activation, positive regulation of phagocytosis, immune response, defense response to bacterium and apoptosis. The primary GO categories for significantly downregulated genes between SS2-infected B6 and control B6 mice were pointed-end actin filament capping(The specific gene involved in this GO was *tmod3*, which was related to movement.), vitamin K biosynthetic process, GDP-L-fucose biosynthetic process, negative regulation of collagen binding, GDP-mannose metabolic process, negative regulation of nucleotide metabolic process, positive regulation of glycolysis, positive regulation of fatty acid biosynthetic process, negative regulation of alpha-beta T cell proliferation and glutamine metabolic process (Fig. 2B). The differentially expressed genes from this study classified into significant GO categories are summarized in Table S2.

### Pathway analysis

**Figure 3:**
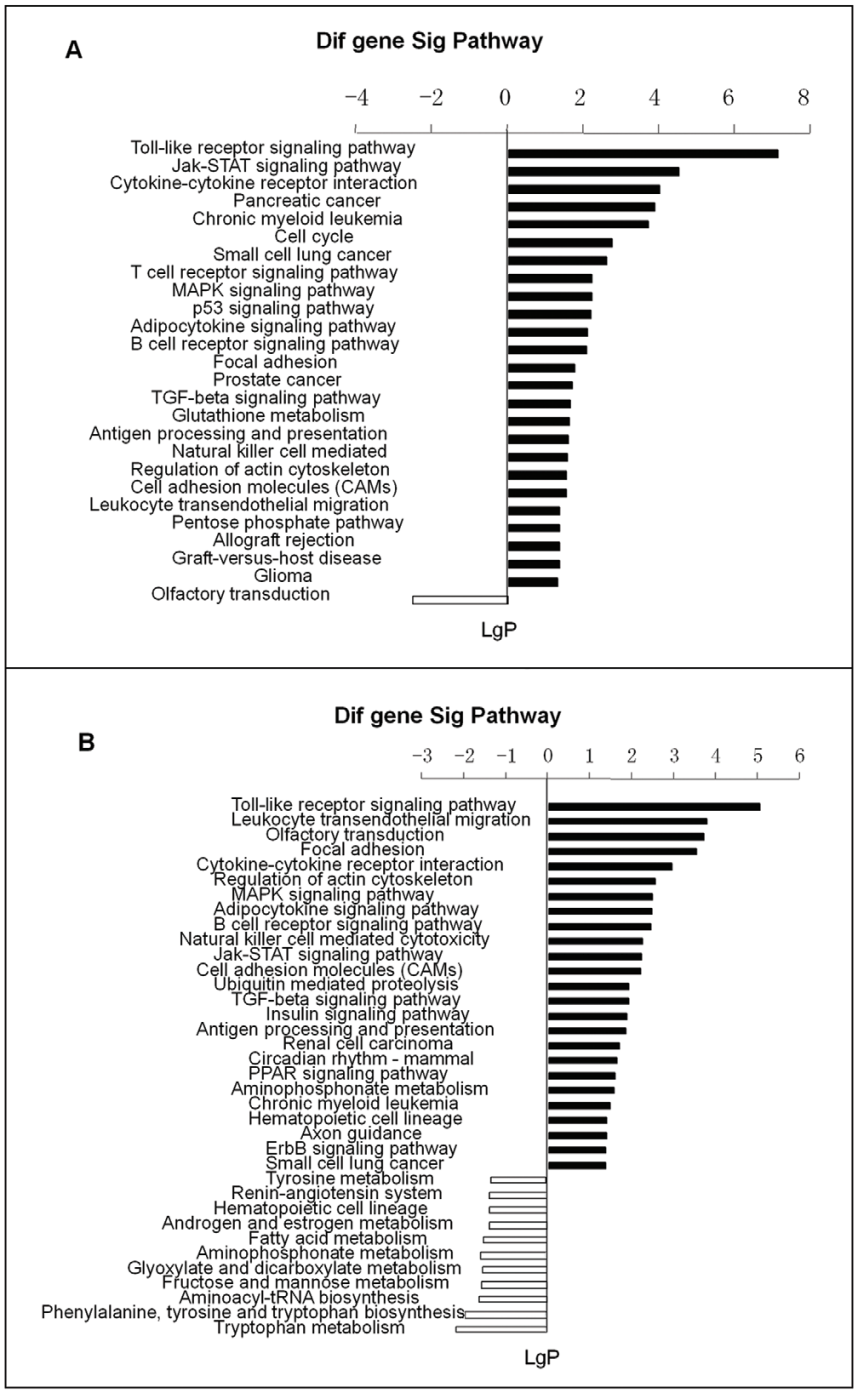
KEGG pathway analysis for significantly differentially expressed genes (A) between SS2-infected A/J and control A/J mice and (B) between SS2-infected B6 and control B6 mice. *P* value<0.05 and FDR<0.05 were used as thresholds to select significant KEGG pathways. LgP is the base 10 logarithm of the *P* value.

The pathway analysis based on the KEGG database was performed on the genes selected as described above. Significantly upregulated genes between SS2-infected A/J and control A/J mice were mainly involved in the toll-like receptor signaling pathway, cytokine-cytokine receptor interaction, T cell receptor signaling pathway, B cell receptor signaling pathway, natural killer cell mediated cytotoxicity, antigen processing and presentation, leukocyte transendothelial migration. Significantly downregulated genes between SS2-infected A/J and control A/J mice were involved in only one pathway, olfactory transduction (Fig. 3A). The KEGG pathway analysis for significantly upregulated genes between SS2-infected B6 and control B6 mice showed that the genes were related to toll-like receptor signaling pathway, leukocyte transendothelial migration, cytokine-cytokine receptor interaction, B cell receptor signaling pathway, natural killer cell mediated cytotoxicity and antigen processing and presentation. The KEGG pathway analysis for significantly downregulated genes between SS2-infected B6 and control B6 mice showed that the genes were related to tryptophan and tyrosine metabolism, phenylalanine, tyrosine and tryptophan biosynthesis, fructose and mannose metabolism, fatty acid metabolism, aminoacyl-tRNA biosynthesis and renin-angiotensin system (Fig. 3B). The differentially expressed genes involved in significant pathways are summarized in Table S3.

### Gene network analysis

**Figure 4:**
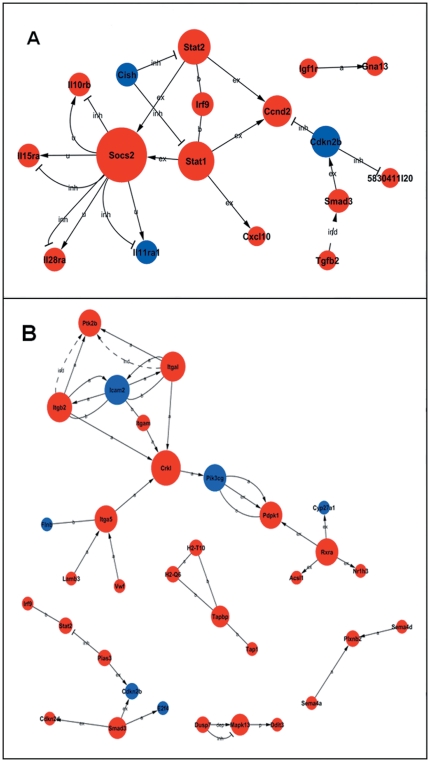
Gene networks of differentially expressed genes involved in significant pathways. The gene networks comprised of the differentially expressed genes involved in significant pathways of (A) A/J mice infected with SS2 and (B) B6 mice infected with SS2 are shown. Legend: each circle represents a gene; red, upregulation; blue, downregulation; line segment, interaction of genes; arrow, activation (a), flat-ended arrow, inhibition (inh); straight, binding (b); dashed line, indirect effect (ind); P, phosphorylation; dp, dephosphorylation; ex, expression; u, ubiquitination.

The differentially expressed genes involved in significant pathways were analyzed for their interaction, and the networks of genes involved in signal transduction during SS2 infection were established utilizing the KEGG database. In the gene network comprised of the differentially expressed genes involved in significant pathways of A/J mice infected with SS2, genes with a high of degree of connectivity, such as *Socs2*, *Sta1*, *Stat2*, were in the core axis of the network. Genes were regulated by their upstream genes when their outdegrees were zero (e.g., *Ccnd2*), or they regulated expression of downstream genes when their indegrees were zero (e.g., *Cish*). The key genes regulated by SS2 infection in the A/J mice were mainly involved in the Jak-STAT signaling pathway and related to cell apoptosis (Fig. 4A, Table 3).

**Table 3:** Degree of key genes in gene network of SS2-infected A/J mice.

Vertex	degree	indegree	outdegree	description
*Socs2*	10	2	8	suppressor of cytokine signaling 2
*Stat1*	6	2	4	signal transducer and activator of transcription 1
*Stat2*	5	2	3	signal transducer and activator of transcription 2
*Irf9*	4	2	2	interferon regulatory factor 9
*Cdkn2b*	3	1	2	cyclin-dependent kinase inhibitor 2B (p15, inhibits CDK4)
*Smad3*	2	1	1	MAD homolog 3 (Drosophila)
*Cish*	2	0	2	Cytokine inducible SH2-containing protein
*Igf1r*	1	0	1	insulin-like growth factor I receptor
*Tgfb2*	1	0	1	transforming growth factor, beta 2
*Ccnd2*	3	3	0	cyclin D2
*Il28ra*	2	2	0	interleukin 28 receptor alpha
*Il15ra*	2	2	0	interleukin 15 receptor, alpha chain
*Il11ra1*	2	2	0	interleukin 11 receptor, alpha chain 1
*Il10rb*	2	2	0	interleukin 10 receptor, beta
*Cxcl10*	1	1	0	chemokine (C-X-C motif) ligand 10
*Gna13*	1	1	0	guanine nucleotide binding protein, alpha 13
*5830411I20*	1	1	0	Data not found

In the gene network composed of the differentially expressed genes involved in significant pathways of B6 mice infected with SS2, some of the genes with a high of degree of connectivity in the core axis were *Icam2*, *Itgal*, *Itgb2*. *Ptk2b* with an outdegree of zero is an example of a gene regulated by upstream genes, while *Rxra* with an indegree of zero represents a gene which regulated expression of other downstream genes.

On the whole, the gene network could be divided into five parts, three of which were related to cell apoptosis in the left top, left bottom and middle bottom of the gene network (Fig. 4B, Table 4). Four genes (*H2-T10*, *H2-Q6*, *Tapbp*, *Tap1*) constituted a small signal transduction network associated with immune responses (center), and three genes (*Plxnb2*, *Sema4a*, *Sema4d*) composed a small nervous system net (bottom right) (Fig. 4B, Table 4).

**Table 4:** Degree of key genes in gene network of SS2-infected B6 mice.

vertex	degree	indegree	outdegree	description
*Icam2*	10	5	5	intercellular adhesion molecule 2
*Itgal*	7	2	5	integrin alpha L
*Itgb2*	7	2	5	integrin beta 2
*Tapbp*	6	3	3	TAP binding protein
*Crkl*	5	4	1	v-crk sarcoma virus CT10 oncogene homolog (avian)-like
*Itga5*	5	3	2	integrin alpha 5 (fibronectin receptor alpha)
*Pdpk1*	5	4	1	3-phosphoinositide dependent protein kinase-1
*Pik3cg*	5	2	3	phosphoinositide-3-kinase, catalytic, gamma polypeptide
*Ptk2b*	4	4	0	PTK2 protein tyrosine kinase 2 beta
*Plxnb2*	2	2	0	plexin B2
*Cdkn2b*	2	2	0	cyclin-dependent kinase inhibitor 2B (p15, inhibits CDK4)
*Rxra*	4	0	4	retinoid X receptor alpha
*Smad3*	3	0	3	MAD homolog 3 (*Drosophila*)

### Confirmation of BeadChips results by qRT-PCR

In order to verify the data obtained by microarray analysis, qRT-PCR was performed. We tested 9 genes differentially expressed between SS2-infected A/J and control A/J mice, and 10 genes differentially expressed between SS2-infected B6 and control B6 mice. As shown in Table 5, the qRT-PCR results largely confirmed the data from the microarray. Notably, the diffscore is the filtering criteria of Illumina for selection of differentially expressed genes. There is no direct relationship with fold change by qRT-PCR. But they have the similar tendency.

**Table 5:** Confirmation of BeadChips results by qRT-PCR.

Gene	Fold change by qRT-PCR	Diffscore by BeadChip
**SS2-infected A/J vs. control A/J**		
*Irf9*	9.3	21.65
*Stat1*	5.46	24.06
*Stat2*	11.22	33.51
*Socs2*	3.18	21.42
*Il10*	6.3	50.89
*Tlr2*	2.71	31.12
*Tnf*	12.21	248.90
*Mmp9*	7.04	90.06
*Ptx3*	34.94	65.08
**SS2-infected B6 vs. control B6**		
*Icam2*	0.48	−30.68
*Itgal*	2.68	34.79
*Itgb2*	2.44	25.06
*Itga5*	3.51	27.16
*Pdpk1*	2.1	28.93
*Stat2*	5.97	39.24
*Tlr2*	1.06	1.86
*Tnf*	2.28	24.82
*Mmp9*	1.99	25.78
*Ptx3*	119	23.02

### Comparison of gene expression

**Figure 5:**
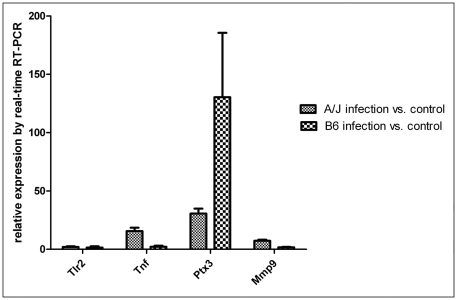
Comparative analysis of gene expression in peritoneal macrophages. Expression levels of *Tlr2*, *Tnf*, *Ptx3* and *Mmp9* in A/J and B6 mice were measured by qRT-PCR and normalized to the housekeeping gene *GAPDH*. Differences between A/J and B6 mice were statistically significant with a *P* value of <0.05 as determined by one-way ANOVA, except with the *Tlr2* gene.

The expression level of toll-like receptor 2 (*Tlr2*) and tumor necrosis factor (*Tnf*) of A/J mice after infection with SS2 were obviously upregulated. There were no changes in *Tlr2* of B6 mice, and the upregulated expression of *Tnf* of B6 mice was significant lower than that of A/J mice after infection with SS2. The pentraxin 3 (*Ptx3*) genes of both A/J mice and B6 mice were upregulated, but its expression level in B6 mice was obviously higher than that of A/J mice. The expression of matrix metalloproteinase 9 (*Mmp9*) in macrophages of B6 mice was lower than that in A/J mice post-infection (Fig. 5).

## Discussion

Gene expression profile analysis was used in this study to identify the candidate genes of susceptibility or resistance to SS2 infection in mice models. While several studies have been performed to evaluate host responses to SS2 infection, this was the first time that the genetic basis of susceptibility to SS2 infection has been studied at the whole transcriptome level.

To confirm host genetic differences in susceptibility to HA9801 infection, A/J and B6 mice were used to determine mortality and clinical signs after infection. We determined that the LD50 of HA9801 in A/J mice was 1×10^7^ CFU between 12 h and 96 h (Table 2), and chose to use just twice the LD50 (2×10^7^ CFU) for subsequent microarray analysis. The inoculated mice showed expected clinical signs of disease such as depression-like behavior, rough appearance of hair coat and swollen eyes [15]. B6 mice injected with a dose of 10^8^ CFU survived and were still active, while a high dose of 10^9^ CFU was required for 100% mortality. The results confirmed that A/J mice were more susceptible to HA9801 infection than B6 mice, consistent with previous research [16].

Several studies have used human or mouse macrophages, porcine choroid plexus epithelial cells (PCPEC), or porcine brain microvascular endothelial cells to determine the host response to *S. suis* infection [27], [28], [29]. We observed several similarities between those reports from *S. suis*-infected cells and our expression profile of SS2-infected mice. For example, we detected the induction of *Mmp9* in peritoneal macrophages in SS2-infected mice, which was also observed in a study using human macrophage cells [27]. Jobin *et al.* showed that whole *S. suis* cells are able to upregulate the production of Mmp9 by human macrophage cells, which may play a critical role in blood brain barrier (BBB) disruption and tissue destruction [27]. Mmp9 is a metalloproteinase that actively counteracts matrix proteins and is secreted by various cell types. Pathophysiological processes characteristic of bacterial meningitis, such as neutrophil extravasation, subarachnoid space inflammation, BBB disruption and brain edema, have all been ascribed to the action of Mmps [30], [31]. Treatment with an Mmp inhibitor has been shown to reduce Mmp9 levels in CSF and significantly attenuate brain damage [31]. Additionally, Mmps have broader functions in the regulation of inflammation and immunity, affecting the activity of cytokines, chemokines and other proteins [32]. In our study, the expression of the gene encoding Mmp9 in SS2-infected A/J mice was increased by 7.04 fold compared with that of control A/J mice, while the fold change was only 1.99 in SS2-infected B6 mice. Therefore, *Mmp9* should be considered a candidate susceptibility gene of A/J mice to SS2 infection. Another example of the similarity between the reports from *S. suis*-infected cells and our transcription profile of SS2 infected mice was the induction of *Tlr2* and *Tnf* in peritoneal macrophages, which were also observed in a study of mouse macrophages [11]. Graveline *et al.* demonstrated that whole encapsulated *S. suis* could influence the relative expression of Tlr2 and further trigger release of Tnf in mouse macrophages [11]. Dominguez-Punaro *et al.* provided evidence that the greater susceptibility of A/J mice was associated with an exaggerated inflammatory response, as indicated by their higher production of Tnf [16]. Here, we observed that fold changes of *Tlr2* and *Tnf* expressions in peritoneal macrophages of SS2-infected A/J mice were 2.73 and 12.2, respectively, while *Tnf* was only upregulated by 2.28-fold, and no change was found in *Tlr2* of SS2 infected B6 mice. Accordingly, *Tlr2* and *Tnf* are candidate susceptibility genes of A/J mice to SS2 infection. Long pentraxin 3 (Ptx3) is a fluid-phase pattern recognition receptor, which plays a non-redundant role in resistance against selected pathogens. With antibody-like functions, Ptx3 is induced by pathogen recognition. It recognizes microbial moieties, activates and regulates complement, and facilitates cellular recognition by phagocytosis [33], [34], [35], [36], [37], [38], [39], [40], [41]. A previous study provided evidence that Ptx3 plays a role in opsonin for internalization of zymosan by mouse peritoneal macrophages [35]. Other lines of evidence have also shown that Ptx3 can regulate inflammatory reactions [42], [43], [44], [45]. For example, Deban *et al.* reported that Ptx3 binds P-selectin and attenuates neutrophil recruitment at sites of inflammation [45]. In our study, *Ptx3* was induced up to 119-fold in peritoneal macrophages of B6 mice after SS2 infection, while the fold change of *Ptx3* was 34.9 in SS2-infected A/J mice. There was no significant difference in expression of *Ptx3* by peritoneal macrophages between control A/J and control B6 mice in BeadChip, while the diffscorebetween SS2-infected A/J and SS2-infected B6 mice was −36.67(Table S1). Therefore, *Ptx3* is a candidate resistance gene of B6 mice against SS2 infection. Together, the studies mentioned above corroborate our findings and provide further validation of our results.

Induction of genes associated with immune responses, inflammatory responses and complement activation is an essential defense mechanism for the host organism, which may help to clear pathogens. Inflammation, a marker of *S. suis* infection, is thought to be responsible for most clinical signs of meningitis, septicemia and sudden death [46]. Dominguez-Punaro *et al.* provided evidence that the greater susceptibility of A/J mice is associated with an exaggerated inflammatory response [16]. Chabot-Roy *et al.* showed that in the presence of specific antibodies and/or complement, *S. suis* may be phagocytosed through different receptors, and that this may result in a faster rate of clearance [47]. In our study, some of the differentially expressed genes (e.g., *Ptx3*, *Fcgr1*) in macrophages between B6 SS2-infected and control mice were involved in positive regulation of phagocytosis. In the gene network composed of differentially expressed genes of B6 mice involved in significant pathways, some genes (*plxnb2*, *sema4a*, *sema4d*) were associated with the nervous system. Expression of these genes may be attributed to clinical signs of meningitis. Apoptosis has been shown to be induced by a wide range of gram-positive and gram-negative bacteria in epithelial and endothelial cells and leukocytes [28], [48]. Therefore, it is plausible that some genes involved in cell apoptosis were upregulated in A/J and B6 SS2-infected mice.

Finally, we observed that muscle-specific gene (*Tmod3*, tropomodulin 3) related to movement, which was involved in pointed-end actin filament capping, was downregulated in SS2-infected B6 mice, potentially reflecting a mechanism in the animal to conserve energy while it combats the bacterial infection. Similar results were obtained in a study by Wu and colleagues that demonstrated muscle-specific genes of zebrafish are downregulated after SS2 infection [49].

The murine macrophage response to SS2 infection showed clear conservation with host responses detected in porcine cells, human cells and other mouse cell or mammalian models. The study produced a set of candidate genes that may influence susceptibility or resistance to SS2 infection in the A/J and B6 mouse models. Among these, *Mmp9*, *Tlr2* and *Tnf* were identified as candidate susceptibility genes of A/J mice and *Ptx3* as a candidate resistance gene of B6 mice against SS2 infection. In future work, we will continue searching for infection markers using these models in order to provide leads for further investigation of *S. suis* pathogenesis.

## Supporting Information

Table S1 (XLS)

Table S2 (XLS)

Table S3 (XLS)
